# Progesterone attenuates temporomandibular joint inflammation through inhibition of NF-κB pathway in ovariectomized rats

**DOI:** 10.1038/s41598-017-15285-w

**Published:** 2017-11-10

**Authors:** Xin-Tong Xue, Xiao-Xing Kou, Chen-Shuang Li, Rui-Yun Bi, Zhen Meng, Xue-Dong Wang, Yan-Heng Zhou, Ye-Hua Gan

**Affiliations:** 1 Department of Orthodontics, Peking University School and Hospital of Stomatology, Beijing, China; 2 Center for Craniofacial Stem Cell Research and Regeneration, Peking University School and Hospital of Stomatology, Beijing, China; 3 Center for Temporomandibular Disorders and Orofacial Pain, Peking University School and Hospital of Stomatology, Beijing, China; 4 Section of Orthodontics, Division of Growth and Development, School 8of Dentistry, University of California, Los Angeles, Los Angeles, California, USA; 5 The Third Dental Center, Peking University School and Hospital of Stomatology, Beijing, China; 6 Precision biomedical laboratory, Liaocheng People’s Hospital, Liaocheng, China

## Abstract

Sex hormones may contribute to the symptomatology of female-predominant temporomandibular disorders (TMDs) inflammatory pain. Pregnant women show less symptoms of TMDs than that of non-pregnant women. Whether progesterone (P4), one of the dominant sex hormones that regulates multiple biological functions, is involved in symptoms of TMDs remains to be explored. Freund’s complete adjuvant were used to induce joint inflammation. We evaluated the behavior-related and histologic effects of P4 and the expression of tumor necrosis factor (TNF)-α, interleukin (IL)-1β, and IL-6 in the synovial membrane. Primary TMJ synoviocytes were treated with TNF-α or IL-1β with the combination of P4. Progesterone receptor antagonist RU-486 were further applied. We found that P4 replacement attenuated TMJ inflammation and the nociceptive responses in a dose-dependent manner in the ovariectomized rats. Correspondingly, P4 diminished the DNA-binding activity of NF-κB and the transcription of its target genes in a dose-dependent manner in the synovial membrane of TMJ. Furthermore, P4 treatment showed decreased mRNA expression of proinflammatory cytokines, and partially reversed TNF-α and IL-1β induced transcription of proinflammatory cytokines in the primary synoviocytes. Moreover, progesterone receptor antagonist RU-486 partially reversed the effects of P4 on NF-κB pathway. In conclusion, progesterone ameliorated TMJ inflammation through inhibition of NF-κB pathway.

## Introduction

Temporomandibular disorders (TMDs) are approximately twice as prevalent (and more severe) in women than in men, similar to rheumatoid arthritis^1^. Sex hormones are reported to be involved in TMD pain^1–3^. Joint inflammation is believed to be a chief cause of pain in patients with TMD^1,4,5^. We and other groups have reported that estrogen aggravates TMJ inflammation or pain through the induction of proinflammatory cytokines in the synovial membrane^6–11^. However, it is still difficult to explain why women with TMD experience some amelioration of pain during pregnancy^12^, even though estrogen increasing throughout pregnancy. Some other hormones may also be involved in TMD pain. Similar to estrogen, progesterone also increase dramatically and steadily during pregnancy^12,13^. Whether progesterone attenuates to TMJ inflammation and pain remains to be explored.

Progesterone is involved in inflammatory response and immune modulating^13,14^. Progesterone decreases interlukine (IL)-6-induced expression of gp130 in hybridoma cells^15^ and inhibits the expression of matrix matalloproteinases in cultured human synoviocytes^16^. Progesterone attenuates rat paw hyperalgesia induced by complete Fruend’s adjuvant. More importantly, progesterone treated mice show decreased arthritis scores and decreased expression of IFN-γ^17^. However, the mechanism underling antiinflammatory effect of progesterone in joint inflammation remains largely unknown.

NF-κB pathway is crucial in the modulation of inflammatory response^17^. Various stimuli and proinflammatory cytokines lead to the nuclear translocation of NF-κB and subsequently promoting transcription of IL-1β, IL-6, and tumor necrosis factor-α (TNF-α)^18^, which appear to be the major proinflammatory cytokines involved in TMJ pathology^19^. Previously, we reported that estrogen-enhanced activation of NF-κB aggravates to TMJ inflammation, and blocking NF-κB attenuates inflammation and pain of TMJ^6^. It has been demonstrated that progesterone decreases cyclooxygenase-2 (COX-2) expression in myometrial cells by inhibits the activity of NF-κB^20^, and a negative interaction exists between the progesterone receptor and NF-κB p65 in both COS-1 and HeLa cells^21^. Whether progesterone inhibits activation of NF-κB in inflamed joint remains to be examined.

In this study, we explored whether progesterone could attenuate TMJ inflammation and pain, and whether progesterone could inhibit NF-κB activity and NF-κB-modulated transcription of proinflammatory genes in the synovial membrane of inflamed TMJs in ovariectomized rats.

## Materials and Methods

### Animals

Adult female Sprague-Dawley (SD) rats were purchased from Vital River Laboratory (Beijing Vital River Laboratory Animal Technology Co., Ltd., China). The experimental protocols were approved by the Animal Use and Care Committee of Peking University and were consistent with the Ethical Guidelines of the International Association for the Study of Pain. Rats were housed on a 12 hours light/dark cycle, under controlled temperature (22 ± 1 °C) and had free access to food and water.

### Progesterone (P4) administration and induction of TMJ inflammation

**Figure 1:**
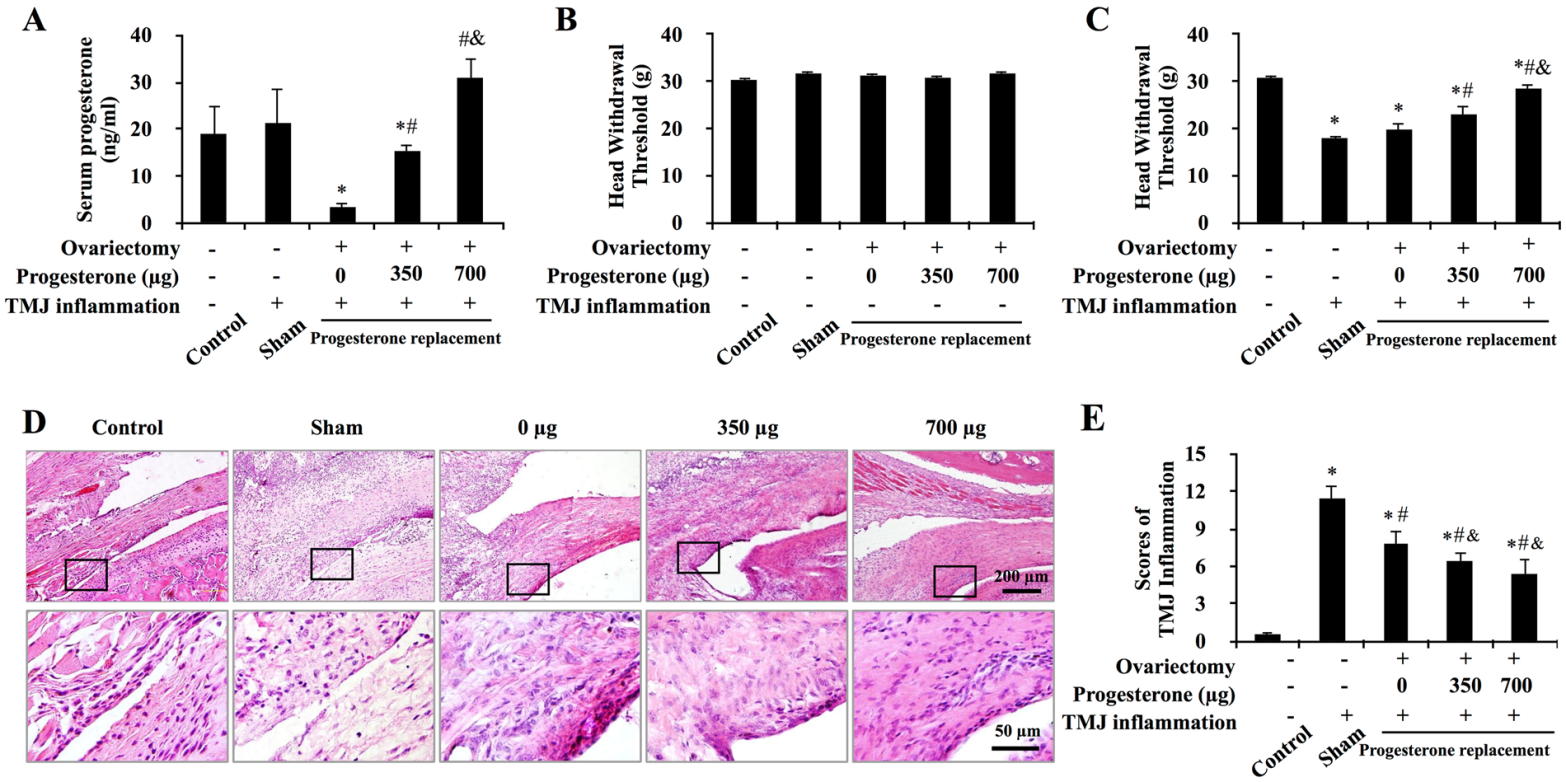
Progesterone attenuated CFA-induced TMJ mechanical hyperalgesia and inflammation. (**A**) Serum levels of progesterone in intact rats and progesterone-treated ovariectomized rats. Among the ovariectomized rats, serum levels of progesterone were lowest in 0 μg-P4 treated rats and increased in a dose-dependent manner with the increasing of injected progesterone. *P < 0.05 versus control and sham; ^#^ *P < *0.05 versus 0 μg; ^&^ *P < *0.05 versus 20 μg, by two-way analysis of variance (ANOVA). (**B**,**C**) Head withdrawal threshold before (**B**) and after (**C**) TMJ inflammation. Progesterone ameliorated the TMJ inflammation–induced decrease in the head withdrawal threshold. (**D**) Representative images of the TMJ regions in female rats. Small and large black frames indicate the original and the magnified areas, respectively. Following induction of inflammation for 24 hours, TMJs of the sham-OVX and the 0-μg-P4 group showed typical features of synovitis. Compared with the 0-μg-P4 group, the proliferation of synoviocytes was less than that in 350-μg-P4 group. In 700-μg-P4 group, the synovial lining layer was thin and infiltration of mononuclear cells around the synovial membrane was not evident. (**E**) Progesterone attenuated CFA-induced TMJ inflammation. Scores for TMJ inflammation decreased with increasing doses of P4. *P < 0.05 versus control; ^#^ *P* < 0.05 versus sham; ^&^ *P* < 0.05 versus 0 μg, by two-way ANOVA.

Scheme for animal experimental was illustrated in Fig. 1A. We randomly divide the rats into 5 groups with 5 rats in each group: control, sham, 0μg-P4, and 350μg-P4, and 700μg-P4 groups. The rats were bilaterally ovariectomized or sham-operated (control and sham rats) after being anesthetized with 1% pentobarbital sodium administered intraperitoneally. One week later, three groups of ovariectomized rats were subcutaneously injected with P4 (Sigma) at doses of 0 μg, 350 μg or 700 μg per rat daily for 10 days, respectively. On the 10th day of P4 treatment, TMJ inflammation was induced by injections of 50 μl complete Freund’s adjuvant (CFA; Sigma) into the upper compartment of bilateral TMJs of ovariectomized and sham-ovariectomized rats. Effectiveness of ovariectomy and P4 replacement were confirmed in Fig. 1A.

### Measurement of head withdrawal threshold

Head withdrawal threshold, which was negatively associated with TMJ nociceptive hypersensitivity, was evaluated as described in detail in our previous study^22^.

### Histology and immunohistochemistry

TMJs were removed *en bloc* and fixed in 4% paraformaldehyde and then decalcified with 10% EDTA (pH 7.4), followed by paraffin embedding. Paraffin sectinos (5 μm) were stained with hematoxylin-eosin and histologically evaluated the TMJ inflammation using the methods described previously^6^. For immunohistochemistry, the paraffin-embedded sections were blocked with 5% BSA and incubated with the primary antibodies against rat NF-κB p65 (1:100; sc-109; Santa Cruz), at 4 °C overnight. After extensive washing with phosphate-buffered saline (PBS), the sections were incubated with horseradish peroxidase-conjugated secondary antibodies and visualized using diaminobenzidine (Zhongshan Golden Bridge Biotechnology, Beijing, China).

### Cell culture and treatments

Fibroblast-like synoviocytes were isolated from synovial membrane of TMJs from 6-week-old rats as previously described^23^, and used for experiment between passages 4 and 6. At the passages used for stimulation, the medium was changed to phenol red-free DMEM/F12 Nutrient Mix (Gibco) containing 15% charcoal-stripped FBS (Hyclone). Synoviocytes were treated with indicated increasing doses of P4 or with combination of 20 ng/ml TNF-α (T 5944; Sigma) or IL-1β (20 ng/ml) (I 2393; Sigma) for 24 hours. Progesterone receptor antagonist RU-486 (100 μM) was added to the media 0.5 hour before treat with P4.

### Immunofluorescent staining

Primary synoviocytes were seeded on 12-well plate slides. After treated by TNF-α and P4 for 24 hours, the cells was fixed with 4% paraformaldehyde, followed by 0.01% Triton-100 treatment. The slides was then blocked by 5% BSA, followed by incubation with antibodies against rat NF-κB p65 (1:100), at 4 °C overnight. After extensive washing with PBS, the slides were incubated with FITC-conjugated secondary antibody (1:200, Jackson ImmunoResearch Laboratories) for 30 minutes at room temperature. Nuclei were counterstained with 4′,6-diamidino-2-phenylindole (DAPI). Confocal microscopic images were obtained using a Zeiss LSM 510 confocal microscopy, and the images were processed using LSM 5 Release 4.2 software.

### Electrophoretic mobility shift assay (EMSA)

EMSA was performed as described in detail previously^6^. Briefly, nuclear extracts were isolated from the TMJ synovial membrane using a Nuclear-Cytosol Extraction Kit (Applygen Technologies Inc., Beijing, China). Single-stranded oligonucleotides containing the NF-κB-binding motif 5′-AGTTGAGGGGACTTTCCCAGGC-3′ (3′ biotin-labeled and unlabeled) and the complementary strand were synthesized. The binding reaction was performed using the LightShift Chemiluminescent EMSA Kit (Thermo Fisher Scientific) in accordance with the manufacturer’s recommendations. The binding samples were separated on a 6% nondenaturing polyacrylamide gel, and membrane-bound probes were incubated with a horseradish peroxidase-conjugated stabilized streptavidin and visualized by enhanced chemiluminescence plus detection (Thermo Fisher Scientific).

### Western blotting

Nuclear proteins from the primary synoviocytes were prepared using a Nuclear-Cytosol Extraction Kit. Equal protein quantities were separated by SDS-PAGE and transferred onto polyvinylidene difluoride membrane. The membranes were blocked by 5% non-fat milk and 0.1% Tween-20 for 1 hour, followed by incubated with NF-κB p65 antibody (1:1000) and β-actin (1:1000, Santa Cruz) antibody overnight at 4 °C. The blots were developed using a horseradish peroxidase-conjugated secondary antibody and enhanced chemiluminescence detection.

### Quantitative real-time polymerase chain reaction (PCR)

Total RNA was extracted from the synovial membrane or cells with Trizol reagent (Invitrogen) under manufacturer’s instructions. RNA samples were reverse-transcribed in a Reverse Transcription system (Bio-Rad). The primers used were as follows: rat β-actin sense/antisense, 5′-TGACAGGATGCAGAAGGAGA-3′/5′-TAGAGCCACCAATCCACACA-3′; rat IL-1β sense/antisense, 5′-CACCTCTCAAGCAGAGCACAG-3′/5′-GGGTTCCATGGTGAAGTCAAC-3′; rat IL-6 sense/antisense, 5′-CCAAGACCATCCAACTCATCTTG-3′/5′CACAGTGAGGAATGTCCACAAAC-3′; rat TNF-α sense/antisense, 5′-CCAGGTTCTCTTCAAGGGACAA-3′/5′-CTCCTGGTATGAAATGGCAAATC-3′^6^. The efficiency of the primers was confirmed by sequencing.

### Statistical analysis

Statistical analysis was performed with SPSS 13.0. All data were presented as mean ± SD. Comparisons between two groups were analyzed using independent two-tailed Student’s t-tests, and comparisons between more than two groups were analyzed using one-way ANOVA. *P* values less than 0.05 were considered statistically significant.

## Results

### Confirmation of ovariectomy and progesterone administration

Serum levels of P4 in the ovariectomized groups received increasing doses of P4 were dose-dependently increased (*P* < 0.05), with which in 0μg-P4 group (3.33 ng/ml) and in 700μg-P4 group (30.73 ng/ml) was the lowest and the highest, respectively. Serum level of P4 in control group (18.91 ng/ml) and sham group (21.31 ng/ml) was higher than that in 0μg-P4 group (*P* < 0.05), comparable with that in the 350μg-P4 group (15.30 ng/ml, *P* > 0.05) and lower than that in the group treated with 700 μg (*P* < 0.05). In addition, serum levels of P4 in all ovariectomized groups remained with the physiologic level of estrous cycle in normal female rats^24^ (Fig. 1A).

### Progesterone attenuated CFA-induced TMJ nociceptive hypersensitivity and inflammation

The head withdrawal threshold was not difference among the groups before induction of TMJ inflammation (Fig. 1B). After induction of TMJ inflammation, the head withdrawal threshold in all the CFA-treated groups were significantly decreased compared with that in the control group (*P* < 0.05). More importantly, TMJ inflammation-induced decrease of head withdrawal threshold was partially reversed by the treatment of P4 in a dose-dependent manner in the ovariectomized-groups compared with that in the sham group (Fig. 1C).

Twenty-four hours after induction of the TMJ inflammation, histology examination showed the feature of synovitis, including proliferation of synoviocytes and infiltration of mononuclear cells around the synovial membrane in the CFA-treated groups, but no such feature was observed in the control group (Fig. 1D). The scores of TMJ inflammation increased significantly in the CFA-treated groups compared with that in the control groups. However, the scores in 0μg-P4 group was lower that of the sham group (*P* < 0.05). More importantly, the scores in ovariectomized groups showed a decreasing trend with the increasing doses of P4 (Fig. 1E).

### Progesterone decreased NF-κB activity in the synovial membrane of inflamed TMJ

**Figure 2:**
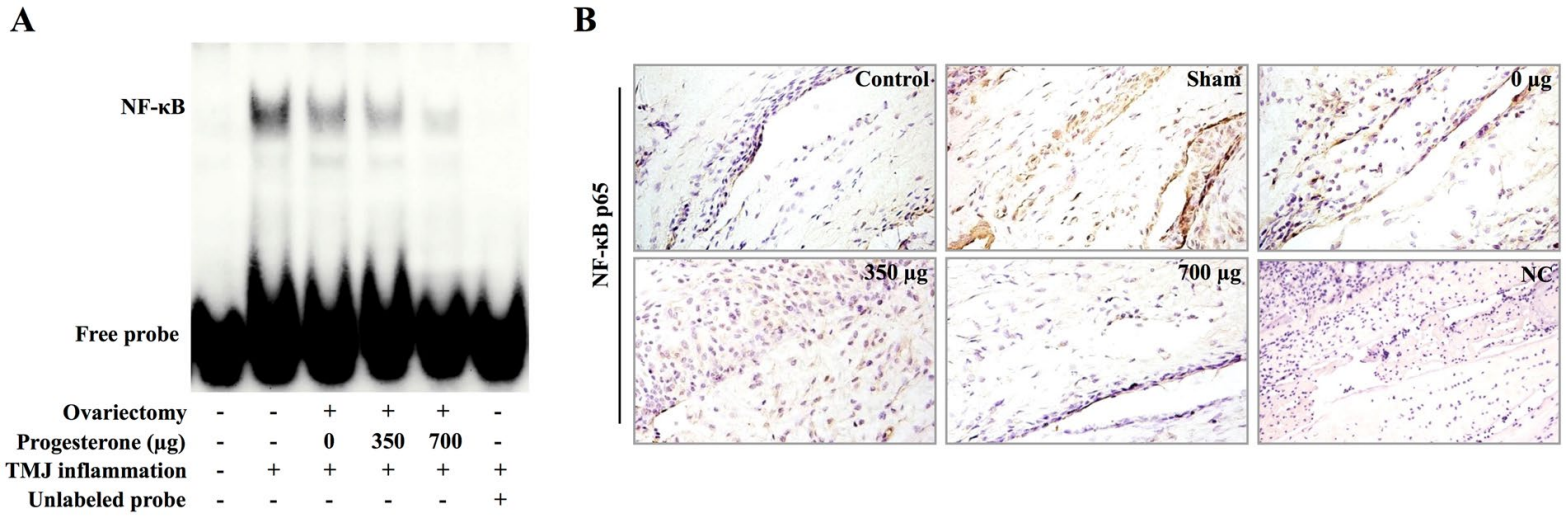
Progesterone decreased NF-κB activity in the synovial membrane of inflamed TMJ. (**A**) Abatement of the DNA-binding activity of NF-κB in the synovial membrane by increasing doses of P4. Activation of NF-κB was examined with electrophoretic mobility shift assay 24 hours after the induction of TMJ inflammation. Unlabeled probes completely blocked formation of the DNA-binding complexes. (**B**) Representative photomicrographs of p65 immunostaining of sections of TMJs 24 h after induction of TMJ inflammation. p65 was rarely expressed in the synovial membrane in control group. After induction of inflammation, p65 was strongly observed in sham group and showed a trend towards decreasing with increasing doses of P4 in the ovariectomized groups.

To evaluate whether P4-induced attenuation of TMJ inflammation was related to NF-κB p65, the DNA-binding activity of NF-κB p65 in synovial membrane was examined by EMSA. As shown in Fig. 2A, after induction of TMJ inflammation for 24 hours, DNA-binding activity of NF-κB p65 was significantly enhanced in the CFA-treated groups compared with that in the control group. However, the activity of p65 induced by TMJ inflammation was dose-dependently reversed in the ovariectomized groups receiving increasing doses of P4. The unlabeled probes of NF-κB completely blocked the formation of DNA-binding complex, indicating that the DNA-binding complex was specific for NF-κB binding sites.

To further confirm the activation of NF-κB in synovial membrane of inflamed TMJ, immunohistochemistry of NF-κB p65 was conducted. It showed that p65 was barely expressed in the synovial membrane of the TMJ from the control rats; after induction of TMJ inflammation, strong expression of p65 was detected in the inflamed synovial membrane of sham-ovariectomized rats (Fig. 2B). However, the inflammation-induced expression of p65 showed a trend of decreasing in the ovariectomized groups receiving increasing doses of P4 (Fig. 2B).

### Progesterone reversed inflammation-induced transcription of NF-κB targeted genes in the synovial membrane of inflamed TMJs

**Figure 3:**
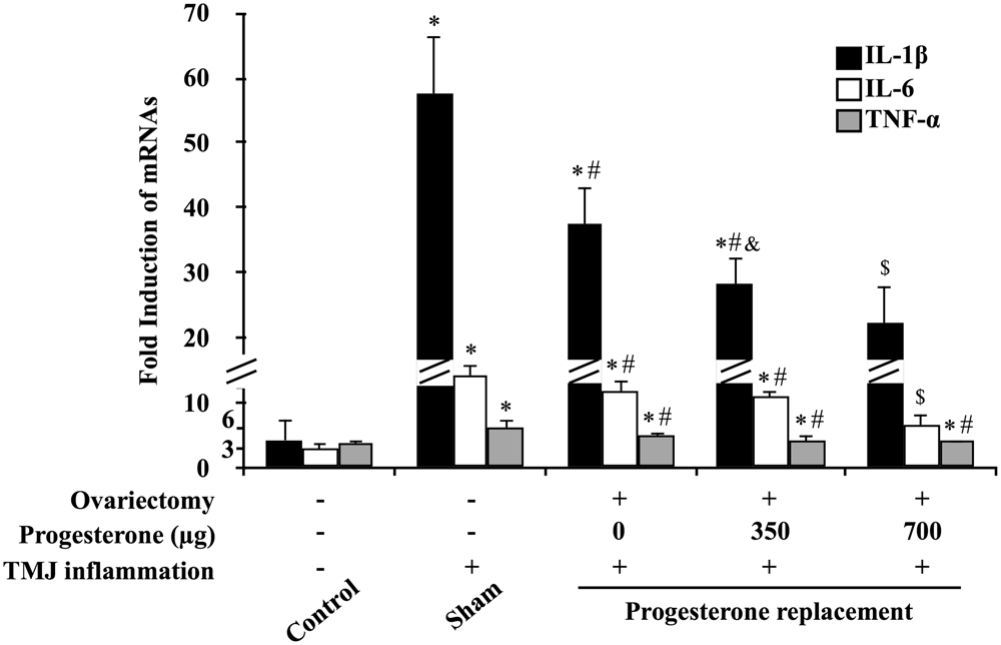
Progesterone decreased transcription of NF-κB target genes in the synovial membrane of inflamed TMJ. Transcription of tumor necrosis factor-α (TNF-α), interleukin-1 (IL-1) and IL-6 in the synovial membrane was examined by real-time polymerase chain reaction 24 hours after the induction of TMJ inflammation. The mRNA expression of these genes was significantly induced in the groups with inflammation compared with control group and was further decreased by P4 in a dose-dependent manner. *P < 0.05 versus control; ^#^ *P < *0.05 versus sham; ^&^ *P < *0.05 versus 0 μg; ^$^ *P < *0.05 versus all other groups, by two-way ANOVA.

The mRNA expressions of NF-κB targeted genes IL-1β, IL-6 and TNF-α were evaluated using real-time PCR. As shown in Fig. 3, mRNA expressions of IL-1β, IL-6 and TNF-α were significantly induced after induction of TMJ inflammation in CFA-injected groups compared with that of the control group. Corresponding to the activity of NF-κB p65, inflammation induced mRNA induction of IL-1β, IL-6 and TNF-α were dose-dependently reversed in the ovariectomized groups receiving increasing doses of P4.

### Progesterone down-regulated transcription of NF-κB targeted genes in primary synoviocytes

**Figure 4:**
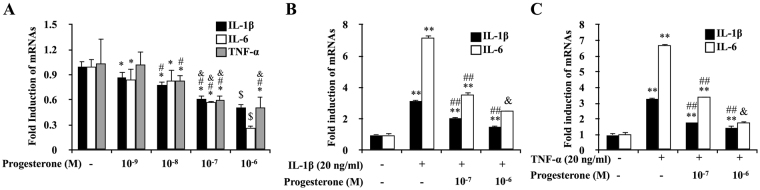
Progesterone down-regulated IL-1β/TNF-α induced IL-1β and IL-6 mRNA expression in FLS. (**A**) Dose-dependent down-regulation of transcription of NF-κB targeted genes by P4 in primary synoviocytes of TMJ. The primary synoviocytes were treated with increasing doses of P4 for 24 h. IL-1β, IL-6 and TNF-α expression was assessed by real-time PCR. *P < 0.05 versus control group; ^#^ *P < *0.05 versus 10-9 M group; ^&^ *P < *0.05 versus 10-8 M group; ^$^ *P < *0.05 versus all other groups, by two-way ANOVA. (**B**,**C**) P4 down-regulated TNF-α/IL-1β induced increasing of IL-1β and IL-6 in primary synoviocytes of TMJ. Expression of IL-1β and IL-6 in the primary synoviocytes treated with P4 and TNF-α/IL-1β alone or both TNF-a and P4 for 24 h, respectively. IL-1β and IL-6 expression was assessed by real-time PCR. *P < 0.05, **P < 0.01 versus control group; ^#^ *P < *0.05, ^##^ *P < *0.01 versus the group treated with TNF-α/IL-1β; ^&^ *P < *0.05 versus all other groups, by two-way ANOVA.

Exposure of the primary synoviocytes of TMJ to increasing doses of P4 for 24 hours resulted in a dose-dependent decrease in mRNA expressions of TNF-α, IL-1β and IL-6 (*P* < 0.05) (Fig. 4A). Furthermore, IL-1β or TNF-α-induced expression of IL-1β and IL-6 mRNA was partially reversed by the treatment of P4 at 10^−7^ μM and 10^−6^ μM for 24 hours (Fig. 4B,C).

### Effect of blocking progesterone receptor on translocation of NF-κB in primary cultured synoviocytes

**Figure 5:**
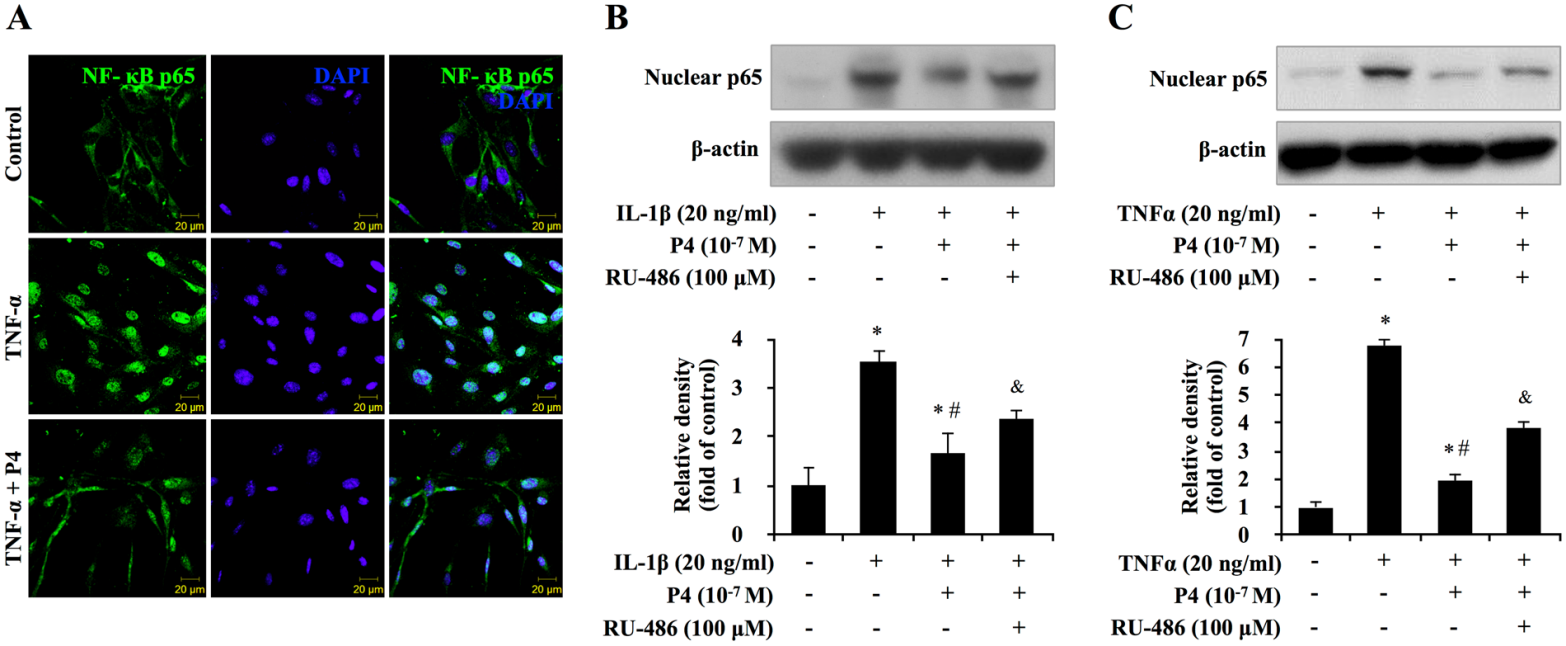
Blocking progesterone receptor on NF-κB p65 nuclear translocation in primary cultured synoviocytes. (**A**) Immunofluorescence of p65 in TNF-α and P4 treated synoviocytes. The subcellular translocation of p65 was examined using confocal microscopy 24 hours after the induction of TNF-α in synoviocytes. Fluorescence signal of p65 (green) was observed in the cytoplasm of the synoviocytes in control group. In contrast, the fluorescence signal of p65 translocated to nucleus in TNF-α-induced group. The green staining of NF-B p65 changed into watery blue after merging with the blue staining of the nucleus, counterstained with 4,6-diamidino-2- phenylindole (DAPI; blue). In TNF-α and P4 treated group, translocation activity was partially blocked by pretreatment of P4 In TNF-α and P4 treated group, with green staining not only observed in nucleus but also in cytoplasm of synoviocytes. (**B**,**C**) Blocking progesterone receptor partially reversed TNF-α/IL-1β-induced transcription of cytokines in primary synoviocytes. The synoviocytes were treated with TNF-α/IL-1β and P4 for 24 h after pretreated with progesterone receptor antagonist RU-486. The expression of p65 was evaluated by western blot or real-time PCR. The middle panels show the quantification of p65 protein presented as the relative density compared with the control group. β-actin was served as an internal control for equal loading. *P < 0.05 versus control; ^#^ *P* < 0.05 versus the group treated with TNF-α/IL-1β; ^&^ *P* < 0.05 versus other groups.

NF-κB is a transcriptional factor and the activated NF-κB locates in the nuclear of the cells. The effect of P4 on nuclear translocation of NF-κB p65 was examined by immunofluorescence. As shown in Fig. 5A, NF-κB p65 was located in the cytosol of the control cells (vehicle), whereas TNF-α stimulated NF-κB p65 to translocate from the cytosol to the nucleus and the pretreatment of P4 partially reversed this translocation.

To further confirm the effect of P4 and progesterone receptor on nuclear p65, western bolt was conducted. As shown in Fig. 5B and C, induction of nuclear p65 induced by IL-1β or TNF-α was partially reversed by the treatment of P4. Moreover, the reversing effect of P4 on nuclear expression of p65 was also partially reversed by pretreatment of progesterone receptor antagonist RU-486, indicating that P4 partially reversing NF-κB p65 nuclear translocation stimulated by IL-1β or TNF-α in the synoviocytes was at least partially dependent on progesterone receptor pathway.

## Discussion

In this study we provide several lines of data regarding that progesterone inhibited attenuated TMJ inflammation and nociceptive hypersensitivity through NF-κB activity in inflamed TMJ. First, progesterone dose-dependently attenuated CFA-injection induced TMJ inflammation and nociceptive hypersensitivity. Second, progesterone repressed NF-κB activity in the synovial membrane of inflamed TMJ and the primary synoviocytes. These finding may help our understanding of the complex roles of sex hormones on joint inflammation and why women experience some amelioration of TMD-related pain during pregnancy.

According to our previous study, estrogen aggravates TMJ inflammation through activation of the NF-κB pathway and the promotion of NF-κB target genes^6^. Ovariectomy also leads to dramatic decreased level of estrogen, thus lead to the repress of inflammation induced activation of NF-κB and the decreased TMJ inflammatory score. In this study, we showed that, although the progesterone level was decreased in OVX group, TMJ inflammatory and NF-κB activation were decreased in OVX group, which was consistent to our previous study. However, further study is still needed for the cross talk between estrogen and progesterone in the process of TMJ inflammation.

Progesterone may ameliorate TMJ inflammation and pain through the repression of synovial inflammation, which is a possible local mechanism underling the effect of progesterone on TMJ pain. Synovial inflammation is one of the major causes of pain for patients with TMD^4,5^. We observed that increasing doses of serum progesterone was associated with decreasing severity of TMJ inflammation and nociceptive hypersensitivity induced by CFA-injection in ovariectomized rats. This phenomenon was further supported by our data that progesterone repressed DNA-binding activity of NF-κB and transcription of NF-κB targeted proinflammatory cytokines IL-1β, IL-6 and TNF-α. Blocking progesterone receptor with RU-486 partially abolished the repressive effect of progesterone on NF-κB nuclear translocation. These data suggested that progesterone could attenuate TMJ inflammation, at least partially through progesterone receptors and NF-κB pathway. Our data also suggested that antiinflammatory effects of progesterone could be one of the reasons for ameliorating TMD pain of women during pregnancy.

The proinflammatory cytokines play an important role in joint inflammation. NF-κB targeted TNF-α, IL-1β and IL-6 appear to be the major proinflammatory cytokines involved in TMJ pathology^19^. Inhibition of these NF-κB targeted proinflammatory cytokines by progesterone provided further details of the antiinflammatory roles of progesterone on TMJ inflammation. In addition to progesterone attenuating expression of these cytokines in the synovial membrane from CFA-injected TMJ, progesterone also partially abolished IL-1β- or TNF-α-induced expression of IL-1β or IL-6 in the primary synoviocytes. More importantly, progesterone alone can repress the basal expression of IL-1β, IL-6 and TNF-α in a dose-dependent manner. These were consisted with the previous study that progesterone attenuates nitric oxide secretion of murine macrophages induced by lipopolysaccharide^25^. Although we previously observed that estrogen can aggravate TMJ inflammation and nociception through NF-κB^6^, it was difficult to explain why women with TMD often experience some amelioration of TMD pain during pregnancy^12^. Based on the observation in the current study, it might help understand to some extent that increasing level of progesterone during pregnancy could antagonize the effects of estrogen, leading to the amelioration of TMD pain in women during pregnancy.

Progesterone played antiinflammatory effect on inflamed TMJ through the modulation of NF-κB pathway. NF-κB plays a pivotal role in several inflammation-associated diseases, such as arthritis, asthma, and inflammatory bowel disease^18,26^. Blocking NF-κB can partially reverse CFA-induced TMJ inflammation^6^. In the current study, we observed that progesterone repressed NF-κB activity in the inflamed synovial membrane, and repressed nuclear translocation of NF-κB p65 in the synoviocytes induced by TNF-α. Moreover, blocking progesterone receptor by RU-486 partially abolished the repression effect of progesterone on NF-κB activity, suggested that progesterone modulated NF-κB through progesterone receptors and its downstream pathway. Our results was consistent with the previous studies that progesterone via progesterone receptor-B inhibits proinflammatory gene expression in human myometrial cells by inhibits the activity of NF-κB^27^, and a negative interaction exists between the progesterone receptor and NF-κB p65 in both COS-1 and HeLa cells^21^. Therefore, progesterone modulating NF-κB activity in the synovial membrane played an important role in ameliorating TMJ inflammation and pain.

Although our results showed that progesterone ameliorate TMJ inflammation and others have shown that progesterone attenuate inflammation-induced thermal hyperalgesia^28^ and nociceptive behavior after excitotoxic spinal cord injury^29^, and that the metabolisms of progesterone were potent sedatives and anaesthetics^30^, there are some researchers reported cycling high level of progesterone does not reduce CFA-induced TMJ nociception^24^, and progesterone even reverses the analgesic effect of estrogen in formalin-induced nociception^31^. These differences indicate that the roles of progesterone are still not fully understood. The complication of the roles of progesterone may be related to the complicated effects of progesterone receptors, for example, increases in the myometrial cell progesterone receptor-A to -B ratio represses the antiinflammatory activity of progesterone receptor-B^27^. Moreover, the combined effects of progesterone and estrogen in rats may differ from the effects of progesterone alone tested in the ovariectomized rats. The ration of progesterone to estrogen may need to be explored to examine the combined effect of the two hormones in the future.

In conclusion, progesterone attenuated TMJ inflammation and nociception through the repression of NF-κB activity and its downstream genes in the inflamed TMJ. These data suggested progesterone might be a new approach for the treatment of TMJ inflammation or pain.

## Electronic supplementary material

Western film for Fig. 5
