# Supplementary material for: Progesterone attenuates temporomandibular joint inflammation through inhibition of NF-κB pathway in ovariectomized rats
Source: Sci Rep. 2017 Nov 10;7:15334. doi: 10.1038/s41598-017-15285-w (PMC5681685; doi:10.1038/s41598-017-15285-w)

Progesterone attenuates temporomandibular joint inflammation through inhibition of NF-κB pathway in ovariectomized rats

Xin-Tong Xue[1,2,†] Xiao-Xing Kou[1,2,†], Chen-Shuang Li[1,2,4], Rui-Yun Bi[3,5], Zhen Meng[3,6], Xue-Dong Wang[1,2], Yan-Heng Zhou[1,2,*], Ye-Hua Gan[3,*]

[1]Department of Orthodontics, Peking University School and Hospital of Stomatology, Beijing, China; and [2]Center for Craniofacial Stem Cell Research and Regeneration, Peking University School and Hospital of Stomatology, Beijing, China; and [3]Center for Temporomandibular Disorders and Orofacial Pain, Peking University School and Hospital of Stomatology, Beijing, China; and [4]Section of Orthodontics, Division of Growth and Development, School 8of Dentistry, University of California, Los Angeles, Los Angeles, California, USA; and [5]The Third Dental Center, Peking University School and Hospital of Stomatology; and [6]Precision biomedical laboratory, Liaocheng People's Hospital, Liaocheng, China.

† Authors contributing equally to this work.
*Co-corresponding authors:
Ye-Hua Gan, DDS, PhD. Center for Temporomandibular Disorders and Orofacial Pain, Peking University School and Hospital of Stomatology, 22# Zhongguancun South Avenue, Haidian District, Beijing 100081, China. Phone:+86-10-82195518; Fax: +86-10-62173402;
Email: kqyehuagan@bjmu.edu.cn
Yan-Heng Zhou, DDS, PhD. Department of Orthodontics. Peking University School and Hospital of Stomatology, 22# Zhongguancun South Avenue, Haidian District, Beijing 100081, China. Phone: +86-10-82195728; Fax: +86-10-82195536;
Email: yanhengzhou@vip.163.com

Supplementary Information

Western film for Fig. 5

B

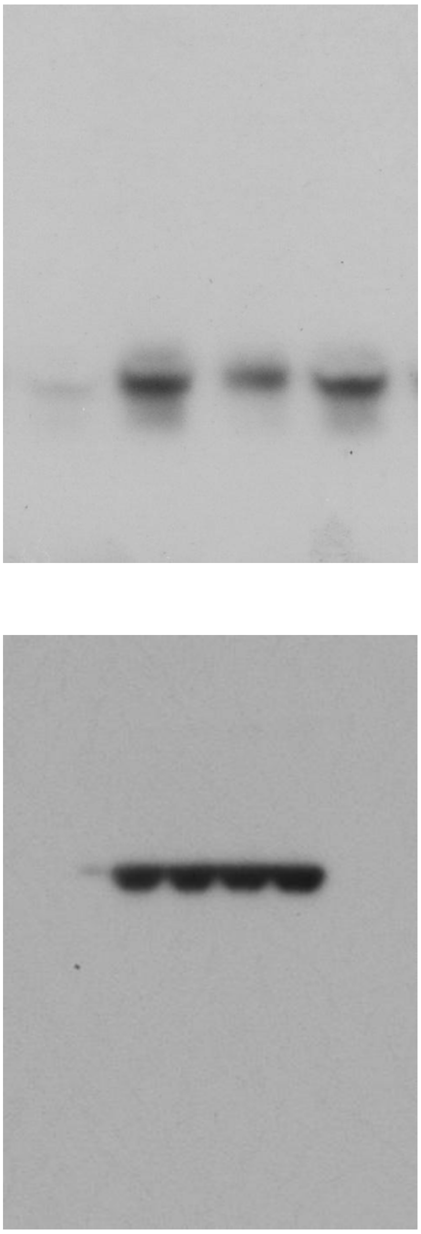

Nuclear p65 **65kD**

β-actin **42kD**

C

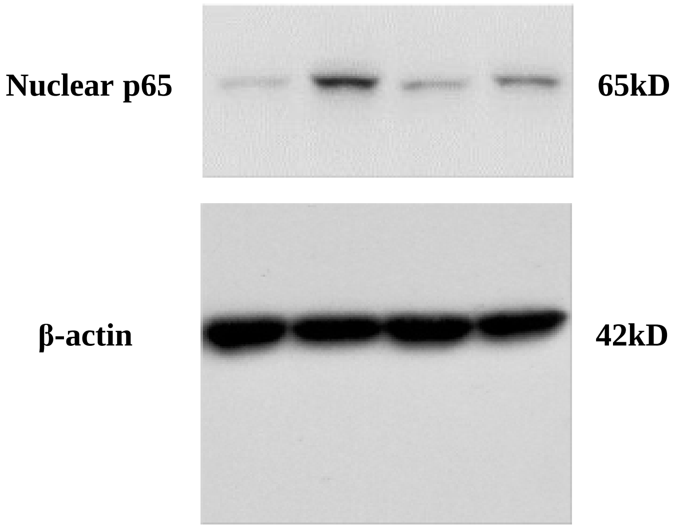

Supplement: Supplementary file 1 — Western film for Fig. 5 [file 41598_2017_15285_MOESM1_ESM.pdf]
